# The Diagnosis of Iliac Bone Destruction in Children: 22 Cases from Two Centres

**DOI:** 10.1155/2016/2131859

**Published:** 2016-08-08

**Authors:** Xiangshui Sun, Yue Lou, Xiaodong Wang

**Affiliations:** ^1^Department of Orthopedics Surgery, Children's Hospital of SooChow University, Suzhou 215003, China; ^2^Department of Orthopedics Surgery, Nanjing Children's Hospital, Nanjing Medical University, Nanjing 210008, China

## Abstract

Iliac bone destruction in children is uncommon and presents various imaging features. Correct diagnosis based on clinical and imaging features is difficult. This research aimed to retrospectively explore the clinical features, imaging, and histopathological diagnosis of children with iliac bone destruction. A total of 22 children with iliac bone destruction were enrolled in this retrospective analysis from two children's hospitals during July 2007 to April 2015. Clinical features, imaging, and histopathological findings were analysed. The mode of iliac bone destruction, lesion structure, and the relationship between the range of soft tissue mass and cortical destruction were determined based on imaging data. The data were analysed using descriptive methods. Of the iliac bone destruction cases, eight cases were neuroblastoma iliac bone metastasis, seven cases were bone eosinophilic granuloma, two cases were Ewing's sarcoma, two cases were osteomyelitis, one case was bone cyst, one case was bone fibrous dysplasia, and one case was non-Hodgkin's lymphoma. Iliac bone destruction varies widely in children. Metastatic neuroblastoma and eosinophilic granuloma are the most commonly involved childhood tumours.

## 1. Introduction

Bone destruction is a form of bone tissue deletion resulting from the replacement of local bone tissue by pathological tissue; this occurs directly as a result of increased osteoclast activity or indirectly as a result of tumour or inflammation, and both the cortical and cancellous bone may be involved. Bone destruction instances with different causes exhibit different imaging characteristics due to differences in the nature of the diseases, the speed of development, and reactive changes of adjacent bones. The imaging features of iliac bone destruction are not as characteristic as those of the long bone, the lesion type varies widely, and the corresponding imaging features exhibit both diversity and similarity [1]. Hence, an accurate diagnosis of bone destruction, especially that caused by malignant tumour, is crucial for designing treatment.

In clinical practice, children presenting with unexplained hip pain or lower limp claudication should receive pelvic digit radiography (DR) including the bilateral hip joints and bilateral ilium [2, 3]. The structure of iliac bone is complex; this bone is the largest irregular flat bone in the body and is located deeply, with unsubstantial iliacus inside and strong gluteus outside. This bone also contains much bone marrow and acts as the largest bone reservoir of bone marrow.

Because iliac bone destruction is uncommon in children, previous studies of iliac bone destruction have often been limited to clinical case reports. Ando et al. reported 3 cases of eosinophilic granuloma of the pelvis in children, and each presented different radiological features and clinical courses [4]. Another case was reported as a diagnostic dilemma, and one of the few documented cases of osteomyelitis presented a nonspecific radiological picture [5]. Because the imaging features vary widely and radiological images differ among cases of iliac bone destruction in children, it is difficult to obtain a correct diagnosis when relying only on clinical and imaging features [6, 7]. Together, these rarities of presentation can result in a diagnostic dilemma, and, in such cases, diagnosis depends mainly on histopathological findings.

This report is a retrospective study that explores the clinical features, imaging results, and histopathological diagnosis of iliac bone destruction in children with the aim of improving the diagnosis of iliac bone destruction in children.

## 2. Materials and Methods

This research is a retrospective analysis of 22 children with iliac bone destruction; the children were recruited from the Children's Hospital of SooChow University and the Nanjing Children's Hospital Affiliated to Nanjing Medical University from July 2007 to April 2015. This research was approved by the Ethics Review Board of the Children's Hospital of SooChow University and the Nanjing Children's Hospital Affiliated to Nanjing Medical University. The study was performed in accordance with the ethical standards described in the 1964 Declaration of Helsinki and its later amendments. No conflict of interests existed.

Clinical features, imaging, and histopathological findings were analysed to improve the diagnostic level of iliac bone destruction in children. The inclusion criteria were as follows: (1) a child patient presenting with hip pain or lower extremity claudication as the initial symptom, and X-ray images showing iliac bone destruction and (2) a routine pelvic radiograph examination in a child patient showing iliac bone destruction without abnormal pathological changes in other organs. The exclusion criteria were as follows: (1) a child patient with previous hematopoietic malignancy, abdominal malignant tumour or soft tissue malignant tumour, and iliac bone destruction; (2) a child patient with iliac bone destruction after suffering severe iliac trauma; and (3) a child with postoperative recurrence of iliac bone destruction with known pathologic diagnosis. The pelvic DR of these patients included the bilateral hip joints and bilateral ilium. Laboratory examinations, including routine blood tests, erythrocyte sedimentation rate (ESR), and the level of alkaline phosphatase, were also recorded. If the pelvic radiograph indicated iliac bone destruction, the patient received a CT or MRI examination to further verify the diagnosis of iliac bone destruction. All patients were diagnosed with iliac bone destruction according to X-ray and CT/MRI examinations were confirmed by operation and histopathological examination. Finally, data were analyzed using descriptive methods.

## 3. Results

Among the 22 patients, the age of onset ranged from 11 months to 12 years (average, 4.9 years), and 14 (63.6%) of the cases were in the 2- to 5-year-old age range. In the studied cases of iliac bone destruction, 6 were on the left side, 13 were on the right side, and 3 were on both sides; 19 cases were found in the iliac ala, and 3 were found in the acetabulum. The study included 15 males and 7 females, with an age range from 11 months to 12 years (average, 4.9 years). The course of the disease ranged from 2 days to 1 month, with an average of 7 days. Among the cases, nine patients were diagnosed with hip joint pain as the first symptom, five were diagnosed with lower limb claudication, four were diagnosed with hip pain and fever, two were diagnosed with backache and malaise, and two were diagnosed unintentionally when X-ray films were recorded for lower limb trauma. Increased ESR was observed in five cases, decreased haemoglobin (lower than 110 g/L) was observed in six cases, increased white blood count was observed in four cases, and elevated alkaline phosphatase levels were observed in four cases.

The imaging feature common to all 22 cases was iliac bone destruction of various forms; 8 cases were accompanied by soft tissue masses of varying sizes, and 2 cases exhibited needle periosteal reaction. Pathology results showed seven disease types: 8 cases of neuroblastoma iliac bone metastasis, 7 cases of bone eosinophilic granuloma, 2 cases of Ewing's sarcoma, 2 cases of osteomyelitis, 1 case of bone cyst, 1 case of bone fibrous dysplasia, and 1 case of non-Hodgkin's lymphoma.

Eight patients with neuroblastoma iliac bone metastasis were from 2 to 6 years old (average, 4.1 years). An X-ray examination showed osteolytic bone destruction without a clear destruction edge in 6 cases and a soft tissue mass in the destruction zone close to the edge of the iliac bone in three cases. Of the 6 patients who received CT scans, 5 showed mixed bone destruction, primarily iliac osteolytic bone destruction; 1 showed osteolytic bone destruction; and 2 showed needle periosteal reaction around the area of bone destruction. Of the three patients who received an MRI examination, the intense signal of fat in the iliac bone marrow cavity was replaced by a weak signal, and soft tissue masses were observed around the lesion, primarily with equal T1 and equal T2 signals, which were uneven.

**Figure 1:**
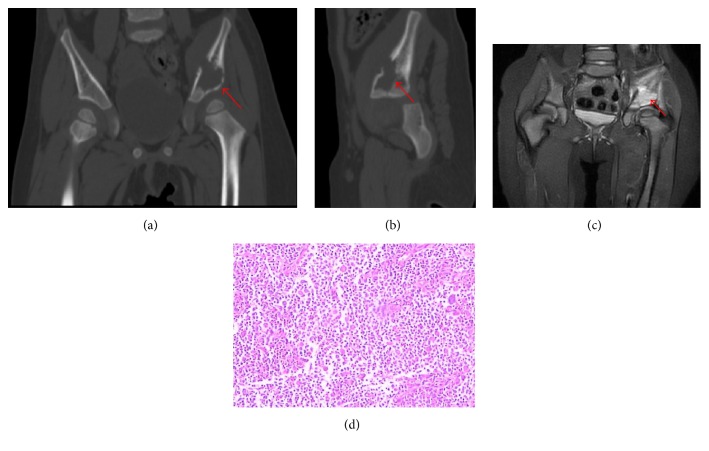
A 2-year-old female patient. The CT results show bone destruction accompanied with periosteal reaction in the left iliac bone, with discontinuous cortical and increased density of the medullary cavity. Surrounding muscle tissue is slightly swollen with a flaky low-density shadow (a, b). MRI shows an expansive destruction and soft tissue mass shadow in the left iliac bone, with equal T1 and slightly long T2 signal (c). The histological appearance of the iliac bone destruction shows a large number of eosinophilic granulocytes invasions (d) (red arrows indicate the site of iliac bone destruction).

Seven patients having bone eosinophilic granuloma were from 2 to 8 years old (average, 4.4 years). The osteolytic bone destruction in these patients was associated with a soft tissue mass shadow of uneven density; the edge was clear, and sclerosis manifestation was not seen. X-ray and CT examinations showed varying degrees of irregular iliac osteolytic destruction; the edge was partly clear, and a soft tissue mass shadow was obvious, with a sclerotic edge in one case. CT examination showed expansive and osteolytic iliac bone destruction zones, an obvious soft tissue mass around the iliac bone, and blurring of the sacroiliac joint interspace (Figure 1).

Both cases of Ewing's sarcoma were in the ilium ala with local irregular osteolytic bone destruction; an obvious medullary cavity sclerosis was observed in one case. Part of the cortical bone was destroyed and absent with rough edges. A large soft tissue mass was seen surrounding the front and back of the abnormal ilium. The ipsilateral sacroiliac joint and hip joint were not involved.

In both cases of osteomyelitis, hemilateral widespread irregular bone destruction was observed with reduced and uneven density, trabecular bone blurring, and varying degrees of sclerotic bone surrounding the lesion. Obvious hyperplasia and sclerosis were often observed around the bone destruction caused by chronic osteomyelitis.

**Figure 2:**
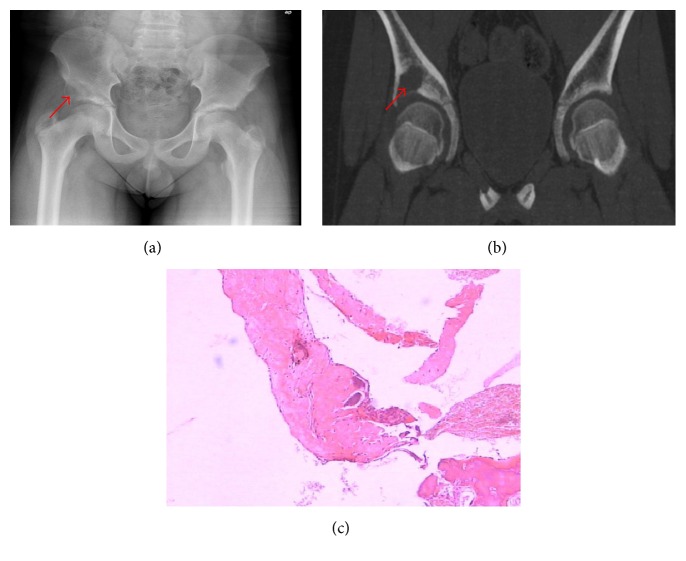
A 12-year-old male patient. X-ray (a) and CT (b) show a bone destruction region with the size of about 34 mm × 32 mm × 29 mm in the anterior inferior margin of the right iliac bone near the acetabulum, with clear edges, local bone expansion, discontinuous cortical, uniform density, and a CT value of 41 HU. The histological appearance of the iliac bone destruction shows that there are fibrous connective tissue and bone tissue (red arrows indicate the site of iliac bone destruction).

One 12-year-old boy had a bone cyst. Single-cystic expansive bone destruction was seen in his right ilium with expansion of the local cortical bone, a clear hardening edge, and discontinuous cortical bone (Figure 2).

One 11-year-old boy had bone fibrous dysplasia. The bone lesion was of the cystic destruction type and presented as an oval-shaped change with a clear boundary, a hardened edge, and expansively thinning bone cortex.

In one patient having non-Hodgkin's lymphoma, the ilium was extensively changed. X-ray and CT examinations of this patient showed worm-eaten osteolytic invasive bone destruction with uneven density around the destruction zone and unclear edge. A mild sclerosis reaction, adjacent soft tissue swelling, and soft tissue mass were observed, and the fat space was blurred. MRI showed T1-weighted images of intermediate signal and T2-weighted images of high signal change in the iliac destruction region and in the muscle around the ilium. The immunophenotype of this case was large B-cell lymphoma.

## 4. Discussion

### 4.1. Clinical Symptoms and Imaging Features of Iliac Bone Destruction in Children

The clinical symptoms of iliac bone destruction in children lack common characteristics. In all 22 cases enrolled in this study, hip pain and lower limb claudication were the most common first symptoms, and a few cases showed fever associated with the first symptom. The course of the disease varied from 2 days to a month, with an average of 7 days. The age of patients mainly ranged from 2 to 5 years (63.6% of all cases). Hip pain was mainly experienced as intermittent dull paint hat was intensified after exercise and relieved after rest, presenting an intermittent attack form [2].

Iliac bone destruction is a form of bone tissue deletion that results from the replacement of local bone tissue by pathological tissue, which occurs directly as the result of increased osteoclast activity or indirectly as the result of tumour and inflammation, and both cortical and cancellous bone may be involved. Bone destruction instances with different causes exhibit different imaging characteristics, due to the differences in the nature of the diseases, the speed of development, and reactive changes of adjacent bones. The imaging features of iliac bone destruction in children are not as characteristic as those of the long bone, the lesion type varies widely, and the corresponding imaging features exhibit both diversity and similarity [1]. Bone destruction is an important sign of the presence of benign or malignant tumours of the ilium in children. Osteolytic bone destruction associated with a soft tissue mass, irregular tumour shape, and vague outline often suggests malignant lesions. The cases of neuroblastoma ilium metastasis and Ewing's sarcoma in this study presented typical osteolytic lesions; however, the edge was unclear, and a soft tissue mass was seen near the edge of the iliac bone destruction zone. The edge may be smooth or blurred, and tumour-associated bone and calcified rings were observed in the lesion, accompanied by a displacement of pelvic organs in large cases. CT scans showed mixed bone destruction, primarily osteolytic bone destruction in most cases, and needle periosteal reaction was observed during operation. MRI examination showed that the intense signal associated with fat in the iliac bone marrow cavity was replaced by a weak signal, and soft tissue masses were observed around the lesion, primarily with equal T1 and equal T2 signals, which were uneven.

Benign lesions causing iliac bone destruction in children also presented as osteolytic destruction, as with the case of bone eosinophilic granuloma (Figure 1) observed in this study. The imaging features associated with benign lesions, such as bone cysts and fibrous dysplasia, mainly presented as cystic or expansive bone destruction with neat tumour morphology, clear outline, sclerotic edge, clear boundary with normal bone tissue, and a remaining bone ridge and hardened edge in some cases, with few soft tissue masses.

### 4.2. Differential Diagnosis

Children presenting with unexplained hip pain or lower limb claudication should receive a pelvic X-ray, including the bilateral hip joints and bilateral ilium [2, 3]. If the X-ray image shows osteolytic iliac bone destruction, the following should be analysed: whether the bone destruction morphology is neat, whether the outline is clear, whether soft tissue shadows break into the lesion, and whether periosteal reaction occurs. CT and MRI examinations can help to differentiate between benign or malignant osteolytic destruction. MRI examination is particularly sensitive to iliac bone destruction associated with a soft tissue mass and is the most sensitive method for visualizing early bone marrow destruction. Auxiliary examination is of little significance for diagnosis, but anaemia often presents in malignant bone destruction [8] with a slightly increased erythrocyte sedimentation rate.

Of the 22 patients with iliac bone destruction, most experienced varying degrees of clinical or radiological misdiagnosis before operation, with the exceptions of one case of osteomyelitis, one case of bone cyst, and three cases of bone eosinophilic granuloma, for which the preoperative diagnoses were more precise. Most of the preoperative misdiagnoses were actually osteomyelitis [9]. Seven disease types were diagnosed by operation and histopathological examination, and the agreement rate between preoperative diagnosis and postoperative pathological diagnosis was 23%. When using imaging, the diversity of pathological types present posed a challenge for qualitative diagnosis and differential diagnosis and increased the requirements for a preoperative radiographic assessment [1].

From a pathological viewpoint, the primary lesion of neuroblastoma lacks specific symptoms and signs and presents a high degree of malignancy. Early metastasis occurs very often in patients with this disease. In 60% of child patients, metastasis has already occurred at the time when they first see a doctor; the most common metastasis sites are the bone and marrow [10]. Because the axial skeleton is rich in red bone marrow, and cancer cells can easily remain here, craniofacial bones, spine, pelvis, and other axial skeleton elements are the most common sites of bone metastasis [8]. The imaging features of neuroblastoma ilium metastasis in children are as follows: (1) osteolytic bone destruction: this is the primary form of bone destruction, presenting as a worm-eaten appearance without sclerotic edge; (2) abnormal bone marrow signal: MRI shows an abnormal signal in the bone marrow cavity, mostly equal T1 signals, and equal or slightly longer T2 signals because normal bone marrow tissue has been replaced by tumour tissue and tumour cells have larger nuclei and less cytoplasm; and (3) irregular mass with bone destruction at the centre: the shape varies greatly; CT often shows equal density, and MRI shows equal T1 signals and equal T2 signals. The pathological changes of bone eosinophilic granuloma mainly present as focal hyperplasia and an aggregation of Langerhans histiocytes in the bone surrounded by eosinophil, lymphocyte, and osteoclast infiltration. The skull is most likely involved, followed by flat bones, such as those of the spine and pelvis, and single bone lesion is the most common form. The imaging features are closely associated with the pathological characteristics. Granulation tissue initiates in the bone marrow cavity, grows expansively, erodes and breaks the cortical bone, and thereby involves the soft tissue; these imaging features and biological behaviour are similar to those of tumours presenting as osteolytic bone destruction. As the disease progresses, cortical bone is penetrated, and granulation tissue is formed, resulting in the formation of a local soft tissue mass [4] (Figure 1). Ewing's sarcoma arises in bone marrow mesenchymal connective tissue; early clinical and radiological diagnosis is difficult, and this tumour is easily misdiagnosed as pyogenic osteomyelitis or another malignant bone tumour [2, 4–6]. The imaging features of this tumour include bone destruction, bone hyperplasia, and periosteal reaction (characterized as “onion skin-like”). The MRI shows an obvious large soft tissue mass surrounding the bone lesion. A definite diagnosis depends on pathological diagnosis [3].

Based on this retrospective analysis of the cases studied here, it can be concluded that neuroblastoma iliac bone metastasis and bone eosinophilic granuloma are the most common forms of iliac bone destruction in children; Ewing's sarcoma, osteomyelitis, bone cyst, bone fibrous dysplasia, and non-Hodgkin's lymphoma are relatively rare [1]. In clinical practice, if children present symptoms such as hip pain or lower limb claudication and the X-ray image shows osteolytic iliac bone destruction, the following should be considered: whether the bone destruction morphology is neat, whether the outline is clear, whether soft tissue shadows break into the lesion, and whether periosteal reaction occurs. Furthermore, CT and MRI examinations should be included in the assessment to more clearly show the location, margin, shape, and density of the lesion as well as the displacement, oppression, or invasion of surrounding structures. Moreover, physicians should be highly alert to the possibility of neuroblastoma iliac bone metastasis in preschool children; therefore, an imaging examination of the retroperitoneum, adrenal gland, posterior mediastinum regions, and other parts is required [10, 11]. Due to the small sample size of this group of cases, the statistical data regarding osteolytic iliac bone destruction might be biased. With an increased sample size, greater changes in the statistical data might be observed.

## 5. Conclusion

Iliac bone destruction in children is comparatively uncommon. This disease type has some characteristic imaging features, but, generally, nonspecific imaging findings were found. A histopathological examination must be considered when making a diagnosis. Neuroblastoma iliac bone metastasis and bone eosinophilic granuloma are the most common forms of iliac bone destruction in children; Ewing's sarcoma, osteomyelitis, bone cyst, bone fibrous dysplasia, and non-Hodgkin's lymphoma are relatively rare.

## Additional Points

1. This study explored the differential diagnosis of 22 children who presented with iliac bone destruction.
2. Metastatic neuroblastoma and eosinophilic granuloma are the most common forms of iliac bone destruction in children.
3. Iliac bone destruction in childhood exhibits some characteristic imaging features, but nonspecific imaging findings were also found. Histopathology results must be considered to obtain a definite diagnosis.
